# Supplementary material for: Chromothripsis during telomere crisis is independent of NHEJ, and consistent with a replicative origin
Source: Genome Res. 2019 May;29(5):737–49. doi: 10.1101/gr.240705.118 (PMC6499312; doi:10.1101/gr.240705.118)
Supplement: Supplemental Material [file supp_gr.240705.118_Supplemental_file_1.zip › contigs/annotated_contigs/DB53/contig.2.DB53_length_349_mean_cov_1.94555873926.docx]

**DB53_length_349_mean_cov_1.94555873926**

```
CCCTTCCCTCCAGAATGGCGACCGAGTGCGACACCCACTAAAACACTA|AAAAATTAAAAAATAAATAACGCACTCCTAAATAGTAGTC
                                                 >chr8:75098269-75098505 - E=1e-109
CCTGTATCTTTCTTTCTCCCCATCTGGATTCAAAATCCTTGGCAGAGCCAGGTGCGGTGGTTCGTGCCTGTAATCTTAACACTTTGGGA

GGACGAGGTGGGCAGATTGCCTGAGCTCAGGAGTTTGAGACCAGCCTGGGCAACAGGGCGAAACCGCATCTCTACTAAAAATATAAAAA

ACTAGCCGGGCA|TGGTGG|CACGCGCCTGCAGTCCCAGCTACTCGGCAGGCTGAGGCTGCAGAATCACTTGAATCTGGGAGGTG             >chr3:139105914-139105985 + E=3e-31
```
